# The Potential of Extracellular Matrix- and Integrin Adhesion Complex-Related Molecules for Prostate Cancer Biomarker Discovery

**DOI:** 10.3390/biomedicines12010079

**Published:** 2023-12-28

**Authors:** Ivana Samaržija

**Affiliations:** Laboratory for Epigenomics, Division of Molecular Medicine, Ruđer Bošković Institute, 10000 Zagreb, Croatia; ivana.samarzija@irb.hr

**Keywords:** prostate cancer, extracellular matrix, integrin adhesion complex, biomarker, tumor stiffness, bone turnover, extracellular vesicles, Proclarix

## Abstract

Prostate cancer is among the top five cancer types according to incidence and mortality. One of the main obstacles in prostate cancer management is the inability to foresee its course, which ranges from slow growth throughout years that requires minimum or no intervention to highly aggressive disease that spreads quickly and resists treatment. Therefore, it is not surprising that numerous studies have attempted to find biomarkers of prostate cancer occurrence, risk stratification, therapy response, and patient outcome. However, only a few prostate cancer biomarkers are used in clinics, which shows how difficult it is to find a novel biomarker. Cell adhesion to the extracellular matrix (ECM) through integrins is among the essential processes that govern its fate. Upon activation and ligation, integrins form multi-protein intracellular structures called integrin adhesion complexes (IACs). In this review article, the focus is put on the biomarker potential of the ECM- and IAC-related molecules stemming from both body fluids and prostate cancer tissue. The processes that they are involved in, such as tumor stiffening, bone turnover, and communication via exosomes, and their biomarker potential are also reviewed.

## 1. Introduction

Prostate cancer is among the leading cancers according to incidence and mortality. In 2020, there were more than 1.4 million new cases of prostate cancer diagnosed worldwide and more than 375,000 deaths from this disease [1]. Prostate cancer is a heterogeneous disease in regard to molecular, morphological, and clinical features. Its course ranges from slow growth throughout the years that requires minimum or no intervention to an aggressive and fatal disease that spreads quickly and resists treatment. Currently, the course and outcome of prostate cancer are unforeseeable, and, therefore, novel biomarkers that will guide prostate cancer diagnosis and treatment are urgently needed. Body fluids are easily accessible compartments that are widely explored in prostate cancer biomarker discovery. However, prostate cancer tissue specimens hold information on the main molecular events and processes that orchestrate tumor growth, progression, and evolution. This information is usually out of reach when other compartments are used. Therefore, the gene and protein expression analyzed from prostate cancer tissue contains an additional wealth of information that could be potentially used for biomarker discovery.

Since the inability to foresee prostate cancer course is one of the main obstacles in its management, it is not surprising that there are numerous attempts in prostate cancer research to deliver the biomarkers of prostate cancer risk, occurrence, risk stratification, therapy response, and patient outcome. Currently, there are more than 46,500 scientific papers on PubMed with the search term ‘prostate AND cancer AND biomarker’ (accessed on 18 November 2023). However, there are only a few biomarkers of prostate cancer, including those stemming from blood, tissue, and clinical characteristics used in clinics. This discrepancy between the number of research items on prostate cancer biomarkers and the number of potential biomarkers that are closely considered to be used in clinics shows how difficult it is to find a suitable biomarker. With such a wealth of information on potential biomarkers, it is hard to review, systematize, and single out those with high potential. Therefore, in this review, the focus is put on the biomarker potential of extracellular matrix (ECM)- and integrin adhesion complexes (IACs)-related genes and proteins stemming from both body fluids and cancer tissue. Given the importance of cell-ECM adhesion for the fate of each cell, ECM and IACs are relevant determinants that potentially hold information content important for prostate cancer initiation and progression.

The ECM is a vital part of the tumor microenvironment that consists of extracellular macromolecules and minerals. The most abundant components of the ECM are collagens, which make up to 90% of the ECM protein. Other common components include fibronectins, laminins, and elastins [2]. ECM was initially considered to be only a structural scaffold without much impact on diverse cellular processes. However, its role in determining almost every characteristic of cell behavior, especially in cancer, is emerging [3,4,5]. ECM proteins are ligands for integrin receptors, and cellular adhesion to the ECM is achieved through their interaction. Integrins are heterodimers that are dynamically expressed on the cell surface [6]. Eighteen known α and eight β integrin subunits exist in humans, and they form twenty-four known heterodimers [7,8]. An important feature of integrin signaling is that upon its activation and ligation, integrin adhesion complexes (IACs) are formed intracellularly [9]. IACs are composed of hundreds of proteins that are collectively named the adhesome [10,11]. Among them, the consensus adhesome has been recognized, which consists of the sixty most common fibronectin-initiated adhesome proteins [12]. Currently, we are witnessing an increasing interest in the role of integrins that encouraged the quest for IACs’ composition (e.g., [13,14,15,16]) and the hierarchy among its components (e.g., [17,18,19]).

Understanding the mechanisms that link the biomarker with the disease increases substantially the informative value of the biomarker and the knowledge of potential obstacles to its use [20]. Therefore, in further sections, the data on the role of ECM-related proteins and integrins in prostate cancer are first presented, followed by the exploration of their biomarker potential in subsequent sections.

## 2. Extracellular Matrix (ECM) and Integrins Influence Prostate Cancer Cell Fate

### 2.1. The Role of ECM Proteins in Prostate Cancer

Matrisome is the collective name for the ECM genes; more precisely, it is the ensemble of genes encoding ECM proteins identified through bioinformatics and the characteristic domain-based organization of the ECM proteins. The matrisome genes are relatively numerous, and the seminal work by Richard Hynes, Alexandra Naba, and colleagues [21,22] recognized more than a thousand such proteins in the human genome, divided into six categories (ECM glycoproteins, collagens, proteoglycans, ECM-affiliated proteins, ECM regulators, and secreted factors). The ECM and the matrisome proteins are involved in almost every feature and process in cancer cells and tissues [23], including proliferation, survival, motility, migration, invasion, epithelial-to-mesenchymal transition (EMT) [24,25], metastasis formation [26], gene expression, response to therapies [2,27,28,29,30], metabolism [31,32], angiogenesis [33,34], and immune system escape [35]. Given that the ECM is the first frontier of the cell towards its surroundings, its involvement in the mentioned processes and the fact that the cells actively respond to their environment are not surprising. Among the most prominent matrisome proteins, the roles of collagens, laminins, matrix metalloproteinases (MMPs), a disintegrin and metalloproteinases (ADAMs), lysyl oxidases (LOXs), cathepsins (CTS), and other diverse glycoproteins and proteoglycans in prostate cancer have been shown. Briefly, collagens, which account for about 30% of the total protein present in the body, are the main structural proteins of the ECM and provide tensile strength to the ECM. The collagen superfamily comprises 28 members numbered with Roman numerals in vertebrates (I–XXVIII) [36]. Generally, collagens are considered to promote tumor growth and create a permissive tumor microenvironment for metastatic dissemination [37]. In prostate cancer, intermediates involved in the biosynthesis and degradation of collagen are widely studied for their potential roles of bone turnover biomarkers linked to prostate cancer bone metastasis (see further text). Along with collagens, laminins are another group of basic ECM and basement membrane glycoproteins that mediate interaction between cells via binding to cell surface receptors (e.g., integrins and dystroglycan) and other components of the ECM. Laminins consist of α, β, and γ chains. There are five α, three β, and three γ chains identified in humans, and they form 16 known different combinations of laminin molecules, named according to their chain composition [38,39]. Changes in the expression levels of some laminins during the progression of prostate cancer have been shown, most notably laminin-332, whose levels were down-regulated [40] in contrast to the unaltered expression of laminin-511 [41]. Laminins are substrates for invading tumor cells; therefore, it is not surprising that, among other roles, they influence the metastatic potential of cancer cells [42]. For example, it was shown that laminin 421, along with type I collagen, type III collagen, and fibronectin, stimulated the migration of prostate cancer cells [43].

The ECM composition is dynamic, and its constant degradation, deposition, and remodeling is an essential part of the processes that include branching morphogenesis, the establishment and maintenance of stem cell niches, wound repair or tissue regeneration, angiogenesis, bone remodeling, etc. Moreover, it is also important for some pathological processes like fibrosis and cancer cell invasion, where it sets the stage for cancer cell dissemination by preparing the trails for their migration. These routes are enriched with released bioactive peptides, growth factors, and cytokines that are otherwise retained within the ECM [44,45,46]. ECM remodeling is highly influenced by specialized proteases with the ability to cleave and degrade the ECM components. They are either expressed on the cell surface or secreted in the extracellular space. It is estimated that approximately half of the 473 active human proteases are expressed in the prostate, of which many are regulated by androgens [47]. To emphasize their importance, the prostate-specific antigen (PSA), which has already been among the most used prostate cancer biomarkers for several decades, also belongs to the kallikrein-related peptidase family (KLK3). Some of the KLKs, including PSA, degrade ECM proteins and activate other ECM-degrading proteases [47,48]. The other most prominent proteases in prostate cancer include MMPs, ADAMs, ADAMs with thrombospondin-1 motifs (ADAMTS), and CTSs. Among them, MMPs are the main enzymes involved in ECM degradation and tissue remodeling, which have been shown in many cases to promote cancer proliferation, invasion, EMT, metastasis, and angiogenesis [49,50]. In prostate cancer, their roles have been discussed in a recent systematic-like review [51], where special emphasis is placed on MMP2, MMP7, and MMP9 since they are the most studied and most often positively correlated with prostate cancer tumorigenicity. Among other roles, MMPs are involved in prostate cancer cell invasiveness (e.g., [52,53,54]), their adhesion and spreading [55], dyscohesion and migration [56], prostate cancer growth in the bone [57], prostate tumor burden, lung metastasis, and blood vessel density [58]. Moreover, recent findings revealed that the androgen receptor (AR), which is among the main drivers of prostate cancer progression, is involved in cross-talk with proteins belonging to the MMP family. Namely, the AR, in interaction with ITGB1 and membrane type-matrix metalloproteinase 1 (MT-MMP-1 or MMP14), activates a protease cascade triggering ECM remodeling, which facilitates the cancer-associated fibroblast invasion through the ECM and a subsequent interaction with prostate cancer cells [59]. ADAMs are another frequent type of proteases involved in ECM remodeling that are also often positively linked to prostate cancer progression. Examples of those are ADAM15, -17, and -28, with mainly consistent literature, and ADAM9, with more complex involvement in prostate cancer (reviewed in [51]). Another group of proteins that increase ECM remodeling and stiffness are the lysyl oxidases (LOX), which influence the alignment of fibrillar collagens and elastins through their crosslinking. Crosslinking transforms soluble collagen and elastin into insoluble, mature fibers. The role of LOX in prostate cancer is controversial [60] since some studies suggest that downregulation of LOX promotes the progression of prostate cancer (e.g., [61]), while other studies have shown that inhibition of LOX enzymes initiated before implantation of AT-1 cells reduces prostate tumor growth. However, the treatment that was started after the tumors were established resulted in unaffected or increased tumor growth [62]. Finally, cathepsins (CTS) are soluble lysosomal or secreted protease enzymes that are also involved in ECM remodeling and shaping the microenvironment [63,64]. In prostate cancer, roles for CTSA [65], -B [66], -D [67], -E [68], -H [69], -K [70,71,72,73], and -L [74,75] have been suggested.

In addition to collagens, laminins, and ECM remodeling proteins, the roles of many other ECM glycoproteins and proteoglycans were implicated in prostate cancer biology. For example, thrombospondins (THBS) are ECM glycoproteins involved in the inhibition of angiogenesis that were shown to regulate the normal prostate in vivo [76], but also to potentiate the cell migration and development of advanced prostate tumors (THBS1) [77] and prostate cancer bone metastasis (THBS2) [78]. Moreover, THBS4 was shown to regulate cancer stem cell-like properties [79].

In conclusion, this brief overview suggests that the ECM and the matrisome proteins largely govern the fate of prostate cancer cells and tissues.

### 2.2. The Role of Integrins in Prostate Cancer

Just like the ECM and the matrisome proteins, integrins are also versatile proteins involved in many cellular and tissue processes. The role of integrins has been excessively documented in prostate cancer. Namely, an early study on the TRAMP (transgenic adenocarcinoma of the mouse prostate) mouse model has shown that integrin β1 deletion enhances the progression of prostate cancer [80]. However, further in vitro studies suggested that integrin β1 in prostate cancer cells switches TGFβ signaling from tumor-suppressive to oncogenic roles [81]. Of the β1 integrin heterodimers, RM11 prostate tumor growth was reduced in the integrin α11-, that is, α11β1-deficient mice [82]. Additionally, integrin α2β1 was shown to decrease proliferation but induce survival and invasion of prostate cancer cells [83]. Further mechanistic insights into this topic revealed that integrin α2β1 inhibition in prostate cancer cells reduces EMT [84]. Importantly, integrin αVβ8 on T cells suppresses anti-tumor immunity in, among others, the TRAMPC2 mouse prostate carcinoma model [85]. This would imply that integrin αVβ8 is a promising target for tumor immunotherapy. However, integrin αV subunit expression was demonstrated to be involved in the acquisition of a metastatic stem/progenitor cell phenotype in prostate cancer [86], confirming the diversity of processes that integrins are involved in. In line with that, integrin β4 was shown to promote the expansion of prostate tumor progenitors [87]. Finally, a recent study suggested that hemidesmosomal α6β4 integrins and plectin are tumor suppressors in prostate cancer but become oncogenic upon hemidesmosome disassembly [88,89].

The role of integrins in prostate cancer metastasis formation has also been documented. Namely, it was shown that prostate cancer metastases from different sites (bone, lymph nodes, and liver) differ in their integrin repertoire [90]. Since the bone is the most common prostate cancer metastatic site, the majority of studies deal with prostate cancer cell communication within the bone. For example, co-expression and enrichment of integrins αV and α5 in osseous metastases were detected [91]. Furthermore, integrins α4 [92] and α4β1 [93] have been implicated in the adherence of prostate cancer cells to the bone marrow endothelium, while integrins α5β1 [94] and α9 [95] were shown to be involved in the interactions of prostate cancer cells with the bone microenvironment. Integrins αVβ6 [96] and αVβ3 [97] were shown to stimulate osteolytic programs and osteoclastogenesis in prostate cancer cells, respectively. Moreover, the roles of integrins α6 [98], α2β1 [99], and β1 [100] have been documented. Finally, an in vitro study on patient-derived xenograft lines suggested that cell-cell adhesion downstream of integrin β1 promoted prostate cancer cells to escape from dormancy when cultured on bone marrow stroma [101].

#### Extracellular Vesicles (EVs)-Derived Integrins Contribute to Prostate Cancer Cell Communication within Its Own Surroundings but also with Other Cells and Tissues within the Body

The role of integrins seems to be important in the process of cellular crosstalk by exosomes and other extracellular vesicles (EVs). EVs are lipid bilayer-encircled particles of nano- to micrometer-scale size that are released into the extracellular environment from almost all types of cells. Exosomes and oncosomes are subsets of EVs. EVs contain nucleic acids, proteins, lipids, and metabolites, and they mediate near- and long-distance intercellular communication [102]. The tumor-derived exosomes from mouse and human lung-, liver-, and brain-tropic tumor cells were shown to have the potential to prepare the pre-metastatic niche when uptaken by organ-specific cells [103]. Treatment with exosomes from lung-tropic models was even able to redirect the metastasis of bone-tropic tumor cells. In the same work, the proteomics analysis of exosomes revealed different integrin expression patterns and their involvement in the process. Namely, the exosomal integrins α6β4 and α6β1 were linked to lung metastasis, and the exosomal integrin αvβ5 was related to liver metastasis. The subsequent work from the Lucia Languino laboratory added substantially to our knowledge of integrins from prostate cancer exosomes and other EVs. Namely, the authors showed that αVβ6 integrin is packaged into exosomes isolated from PC3 and RWPE prostate cancer cell lines and transferred to and internalized by DU145 αVβ6 negative cells, which enhances their ability to adhere and migrate [104]. M2 polarization and angiogenesis were also shown to be affected by the EV transfer of αVβ6 from prostate cancer cells to monocytes and endothelial cells, respectively [105,106]. Furthermore, the αVβ3 integrin is also expressed in exosomes released from PC3 and CWR22Pc prostate cancer cells, and its exosome-mediated transfer from tumorigenic to nontumorigenic BPH-1 cells promoted their migration [107]. A single treatment with small EVs released from prostate cancer C4-2B cells that express αVβ3 stimulated tumor growth and induced differentiation of prostate cancer cells towards a more aggressive neuroendocrine phenotype [108]. To add to the importance of these events, the same authors have shown that prostate cancer sheds the αVβ3 integrin in vivo through exosomes [109]. The role of β1 integrins was also demonstrated; namely, small EVs from either cancer cells or blood from tumor-bearing TRAMP mice transfer β1 integrins, which stimulate anchorage-independent growth of recipient cells [110]. Other authors have also demonstrated the involvement of integrins from EVs in prostate cancer. Specifically, it was shown that a subline of prostate cancer DU145 cells that is resistant to inhibitors of the mevalonate pathway releases large oncosomes, which are atypically large EVs. Large oncosomes from these cells overexpress integrin αV and promote adhesion and invasion in recipient DU145 parental cells [111]. Finally, exosome-mediated transfer of integrin α2 derived from castration-resistant prostate cancer cells promotes migration and invasion of androgen receptor-positive prostate cancer cells by inducing EMT [112]. Taken together, these results suggest that EV-derived integrins have important roles in the communication of prostate cancer cells with their near and distant environment.

**Figure 1:**
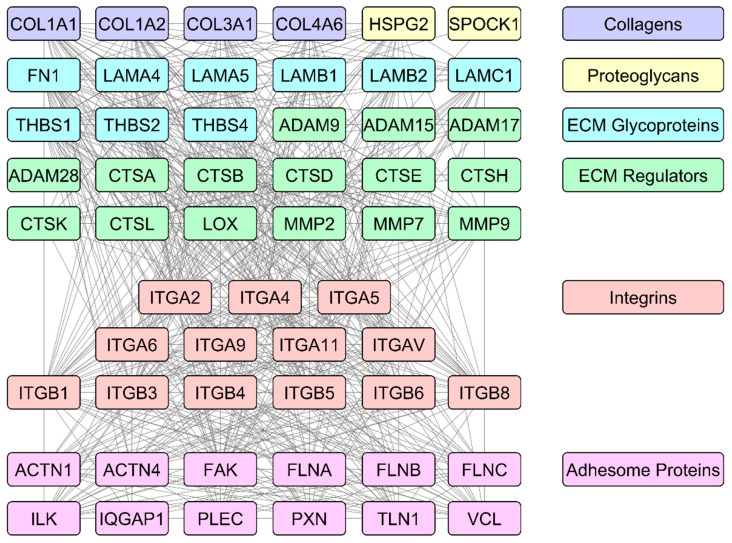
Protein–protein interaction network of a selection of ECM and IAC proteins with suggested roles in prostate cancer. The list is not exhaustive, and the most prominent ECM core proteins, ECM regulators, integrins, proteins of consensus, and the integrin αVβ5 adhesome were considered. The network was retrieved from the STRING database (version 12.0) [113] and visualized in Cytoscape (version 3.10.1) [114].

The main drivers of prostate cancer progression from the repertoire of ECM and IAC-related proteins and their interactions are shown in Figure 1. Given the essential roles of ECM and IACs in prostate cancer biology, it is to be expected that their genes, proteins, and the processes they are involved in would show high potential for biomarker discovery. Those possibilities are explored in more detail in further sections.

## 3. Increased Tumor Stiffness Enables Prostate Cancer Detection Via Physical Palpation

A frequent characteristic of different tumor types, including those of the lung, breast, colon, pancreas, and liver, is increased tumor stiffness, which is a consequence of excessive deposition of ECM components, especially type I collagen. Among the properties that are affected by increased tumor stiffness are tumor initiation, growth, invasion, metastasis, immune cell infiltration, stem cell differentiation, growth factor secretion and signaling, and angiogenesis and vessel permeability [115]. In prostate cancer, increased tumor stiffness has also been documented and correlated with more malignant tumors with a higher Gleason score (reviewed in [115]). Signaling pathways activated by ECM stiffness and linked to castration resistance mechanisms in prostate cancer have been recently reviewed [115]. Briefly, they involve AR-centric (for example, the expression of constitutively active AR variants) and AR-independent mechanisms, which might include the activation of FGFR or modulation of the WNT pathway to drive AR-independent growth of prostate cancer. Among other malignant characteristics, matrix stiffness has been recently shown to induce EMT in prostate cancer cells [116]. Furthermore, it was shown that prostate cancer PC3 cell adhesion to endothelial cells increased with matrix stiffness, suggesting that tumor matrix stiffness promotes cancer cell interaction with the endothelium, which is among the key steps in cancer cell intravasation and dissemination within the vasculature [117]. Additionally, prostate cancer cells cultured on stiff substrates have higher migration potential [118]. Finally, prostate cancer cell contractility, viscoelasticity, and modulation of cellular stiffness are also differentially influenced by substrate stiffness [118,119]. Taken together, these brief outlines suggest that tumor stiffness largely determines the fate of prostate cancer cells.

Because of increased stiffness, prostate cancer lesions feel harder upon physical palpation than the surrounding, healthy tissue. Consequently, this characteristic enables prostate cancer detection through physical palpation in a digital rectal examination (DRE). According to the most recent European Association of Urology guidelines [120,121], the cT staging of prostate cancer is based on DRE only. The cT staging, which is a part of the TNM (tumor, node, and metastasis) classification, is used to determine the clinical stages of the tumor, that is, to describe the size of the tumor and how far it has grown. However, DRE used alone has sensitivity and specificity below 60%, so it cannot be considered to exclude prostate cancer [122]. Although PSA levels in the blood are a better predictor of prostate cancer [121], an abnormal DRE is still an indicator for prostate cancer biopsy and adds significantly to its diagnosis.

## 4. Bone Turnover: ECM Biomarkers in Prostate Cancer Bone Metastasis Diagnosis and Prognosis

It is estimated that about 70–90% of prostate cancer patients with advanced disease will develop bone metastasis (usually homed in the axial skeleton of the pelvis or spine) that is frequently accompanied by intolerable bone pain that lowers life quality and impairs overall survival [123,124]. However, bone metastases are challenging to detect before their final stages. Tissue biopsies and imaging are used in the diagnosis of bone metastasis, but these methods are expensive, highly invasive, with low specificity and sensitivity, and with the risk that is brought about by radiation exposure. Therefore, novel diagnostic and prognostic biomarkers are needed. Since prostate cancer cell growth in the bone metastatic site is accompanied by bone remodeling, the bone formation and bone resorption biomarkers are promising molecules for the diagnosis and prognosis of prostate cancer bone metastasis, which is primarily characterized by osteoblastic lesions, that is, uncontrollable bone formation. The value of bone turnover biomarkers is especially emphasized by the fact that bone turnover releases specific molecules in the blood and urine.

Bone turnover markers include several molecules from the pathway of collagen biosynthesis and degradation. Namely, pro-collagen type I N-terminal pro-peptide (PINP) and pro-collagen type I C-terminal pro-peptide (PICP) are the collagen-related bone formation biomarkers that contribute to the production of mature type I collagen. On the other hand, C-telopeptide of type I collagen (CTx), N-telopeptide of type I collagen (NTx), and C-terminal pyridinoline cross-linked telopeptide of type I collagen (ICTP) are bone resorption biomarkers released in the process of collagen breakdown. Furthermore, pyridoxine (PYD) and deoxypyridinoline (D-PYD) cross-links help to keep collagen fibers stable and are found within and between collagen molecules. They are also released into the blood upon the breakdown of bone tissue. The biomarker potential of PINP, PICP, CTx, NTx, ICTP, PYD, and D-PYD in prostate cancer has been extensively reviewed [123,124,125,126,127,128,129]. Briefly, bone metastases were repeatedly shown to increase PINP and PICP levels in the serum of prostate cancer patients [123], and the rise in levels of PINP in the serum is a sign of prostate cancer bone metastasis progression [130]. Furthermore, PINP was correlated with survival in prostate cancer patients with bone metastasis [131,132]. Increased blood levels of PYD, D-PYD [123], and ICTP [130] were also found in individuals with prostate cancer bone metastasis. Higher PYD levels were linked to worse overall survival [133], while ICTP was shown to be a useful biomarker for prostate cancer bone metastasis, especially when combined with other biomarkers [134]. Although some earlier studies suggested that NTx and CTx had a low sensitivity for diagnosing prostate cancer bone metastasis [135], a recent meta-analysis on NTx revealed that the pooled sensitivity and specificity for metastasis detection were 77% and 80%, respectively, for 45 diagnostic studies with combined cancer types that included prostate cancer [136]. Additionally, the prognostic value of NTx in human cancers with bone metastasis revealed that high NTx levels were linked to poor overall survival. Further to this, another recent study suggested that high levels of bone turnover biomarkers were associated with worse overall survival in men with newly diagnosed metastatic prostate cancer [137]. Taken together, these studies suggest that bone turnover proteins from the pathway of collagen synthesis and breakdown have biomarker potential in prostate cancer bone metastasis diagnosis but also bear prognostic value since they are correlated with the survival of prostate cancer patients. Other bone formation (BGLAP (osteocalcin) and SPP1 (osteopontin)) and bone resorption (IBSP (integrin-binding sialoprotein or bone sialoprotein)) ECM glycoproteins were also explored for their biomarker potential in prostate cancer metastasis formation and are discussed in more detail in [123]. Briefly, further research is required to define their potential biomarker roles.

## 5. The Biomarker Potential of EVs-Derived Integrins in Prostate Cancer

The EVs are accessible by minimally invasive or non-invasive methods and are widely studied [138] in search of cancer biomarkers (reviewed in [139,140,141,142,143,144]). In prostate cancer, the exosomes from different compartments were studied [145]. For example, the potential of urinary EVs [146] and plasma-derived exosomes [147] was explored. In a recent article, the authors did the proteomic analysis of EVs isolated from the blood sera of prostate cancer patients, healthy donors, patients with hypertrophic disease, and post-prostatectomy disease-free cases. The antibody-based proteomic technology analysis (reverse-phase protein microarrays) showed that integrin β5 is among the few differentially expressed proteins in tumor-derived EVs with diagnostic potential [148]. In a previously mentioned study [111], based on in vitro and in vivo prostate cancer models and patient tumor tissues, it was suggested that αV integrin-positive large oncosome-like structures were present in tumor xenografts and prostate cancer patients’ tissues. Moreover, increased αV-integrin expression in tumors was correlated with a high Gleason score and lymph node status. Furthermore, in a study based on a PC-3 cell model, it was suggested that exosomal integrin β4 and vinculin were potential biomarkers for the progression of prostate cancer associated with taxane resistance since their expression was upregulated in exosomes derived from PC-3R taxane-resistant cells compared to PC-3 parental cells [149]. Finally, exosomal integrin α3 was shown to interfere with non-cancerous prostate epithelial cells’ functions. The western blot analysis from the same study performed on urine samples from 13 patients in total has revealed that integrins α3 and β1 were more abundant in the urine exosomes of metastatic patients compared to benign prostate hyperplasia or prostate cancer [150]. In conclusion, besides their functional role in prostate cancer, the EVs-derived integrins are also a source of potential prostate cancer biomarkers.

## 6. ECM- and Integrin Adhesion Complex (IAC)-Related Single Genes and Proteins as Prostate Cancer Biomarkers

While increased tumor stiffness, bone turnover, and the priming of metastatic niches are complex processes involving a larger group of proteins and a series of steps, the role of individual ECM- and IAC-related genes and proteins and their biomarker potential in prostate cancer has also been extensively documented. Matrisome proteins are grouped in the core matrisome and matrisome-associated protein categories [21,22]. For the purposes of this review article, the core matrisome proteins (n = 274) were examined for their potential roles in prostate cancer biomarker discovery. Additionally, MMPs, ADAMs, LOXs, and CTSs from the ECM regulators subcategory of matrisome-associated proteins are included because of their important roles in ECM remodeling, which is especially emphasized during cancer progression. Table 1 summarizes the information on the biomarker potential of single ECM-related genes and proteins in prostate cancer. Although the list of genes, proteins, and publications in Table 1 is not exhaustive, Table 1 already refers to more than two hundred articles, which tells about how extensively the ECM proteins were studied in prostate cancer. Among the most studied genes and proteins from Table 1 are IGFBP1 (insulin-like growth-factor-binding protein 1), -2, -3, CYR61 (cysteine-rich angiogenic inducer 61), POSTN (periostin), and SPON2 (spondin 2). Insulin-like growth factors (IGFs) are related to insulin but have much higher growth-promoting activity. Approximately 98% of IGF1 is always bound to one of six binding proteins (IGFBPs). IGFBP3, the most abundant among them, accounts for 80% of all IGF binding. Binding to an IGFBP increases the half-life of IGF in the circulation and blocks its potential binding to the insulin receptor, which regulates IGF signaling [151]. One mechanism for regulating the biological activity of IGFBPs is their differential localization to the cell surface, or ECM [152]. While the biomarker potential of IGFBP2 and -3 in prostate cancer is a matter of reports emphasizing its complexity (see references from Table 1 and [153,154,155,156,157,158,159,160,161]), the articles on the roles of IGFBP1 are scarcer but more unison, suggesting its potential role as the biomarker of prostate cancer risk, disease stratification, and prognosis (Table 1). Just like the IGFBPs, CYR61, POSTN, and SPON2 also belong to the category of ECM glycoproteins. While the articles on SPON2 (Table 1) mainly suggest its diagnostic potential in prostate cancer, the literature on the other two proteins additionally explores their potential roles as prognostic, predictive, and risk stratification biomarkers. Among them, CYR61 is a secreted growth factor-inducible protein that promotes adhesion and interacts with several integrins. It plays a role in cell proliferation, differentiation, apoptosis, angiogenesis, wound healing, and extracellular matrix formation. Earlier studies on prostate tissues [162] and sera from prostate cancer patients [163] suggested that there was a strong association between CYR61 expression and prostate cancer and that CYR61 expression was correlated with more advanced disease (Gleason score of 8 or greater) and non-organ-confined in comparison to organ-confined prostate cancer. However, decreased expression of CYR61 in cancer tissues is associated with prostate cancer recurrence after surgical treatment [164], suggesting that CYR61 expression increases during prostate cancer development and decreases with further disease progression [165]. POSTN is a secreted ECM protein that induces cell attachment and spreading and plays a role in cell adhesion, tissue development, and regeneration. Earlier studies dealt with the risk stratification potential of POST expression in prostate cancer tissues and stroma and suggested its association with the Gleason score, the stage of the tumor, the degree of malignancy, and bone metastasis [166,167,168,169]. Further proteomics study found significant up-regulation of POSTN in prostate cancer tissue in comparison to benign prostate hyperplasia [170], confirming its diagnostic potential. Finally, other articles established that POSTN stromal and epithelial expression on prostate cancer biopsy samples has a prognostic value since it is correlated with the prostate cancer patients’ clinical outcome [171,172,173,174,175,176]. Other frequently studied ECM glycoproteins in prostate cancer include ADIPOQ (adiponectin), IBSP (integrin-binding sialoprotein), SPARC (osteonectin or secreted protein acidic and cysteine-rich), and SPP1 (osteopontin). Among them, IBSP and SPP1 were already mentioned in Section 4 for their roles as potential bone metastasis biomarkers. However, they are studied more extensively than that, as evident from Table 1.

Since ECM remodeling plays an essential role in cancer cell dissemination, it is not surprising that its main executors are widely explored for their biomarker potential. A recent systematic-like review [51] presented published literature on the roles of the metzincin superfamily of proteases in prostate cancer. The vast majority of reviewed papers can be considered to deal with the biomarker potential of MMPs, TIMPs (tissue inhibitors of metalloproteinases), ADAMs, and ADAMTS, since many of those are correlated to prostate cancer and/or its clinical stages and progression. The number of papers that deal with their biomarker potential in prostate cancer exceeds the amount of literature published on other groups of genes and proteins in Table 1. In Table 1, MMP2 and MMP9 stand out because their biomarker potential has been frequently studied and documented in the literature. Namely, it was suggested that MMP2 has diagnostic, prognostic, risk stratification, and risk of disease biomarker potential in prostate cancer (Table 1). Moreover, an interesting paper found that the expression of MMP2 in bone marrow micro-metastasis is associated with the presence of circulating prostate cells and a worse prognosis in prostate cancer patients treated with radical prostatectomy [177]. For MMP9, its potential diagnostic, prognostic, and risk stratification biomarker values were suggested (Table 1). However, it should be noted that not all studies on MMP2 and MMP9 (and MMPs in general) in prostate cancer are unison (discussed in more detail in [51]). For example, the MMP9 prognostic and risk stratification potential were questioned since different studies came to different conclusions (e.g., [178,179]). Of the articles that studied a group of MMPs and TIMPs, a recent paper showed that the MMP gene expression signature consisting of MMP1, MMP7, MMP11, MMP24, and MMP26 had a high prognostic value since patients with a high MMPS risk score and low M2 macrophages showed the worst recurrence-free survival [180]. Finally, another recent article suggested that MMPs/TIMPs expression in biopsy tissues may reflect evolutionary changes from prostate benign tissues to prostate cancer and add to its diagnosis [181]. However, speaking in general, the role and the biomarker potential of TIMPs in prostate cancer are frequently the subject of conflicting reports (reviewed in [51]). Among ADAMs, ADAM9, -10, -12, -15, -19, and -28 (Table 1) were suggested to have biomarker potential in prostate cancer. The role of LOX as a prostate cancer biomarker is complex, since one study found that high LOX expression in the non-malignant prostate epithelium predicts a poor outcome in prostate cancer patients that are managed by watchful waiting. The same study found that the LOX expression score in prostate tumor epithelium was positively correlated to the Gleason score and metastases but was not associated with cancer survival [182]. However, another study suggested that low LOX expression in tumors detected by immunohistochemistry was associated with a poor prognosis in patients with prostate cancer [183].

The literature on the biomarker potential of laminins in prostate cancer is less abundant than in other cancer types (e.g., [184]). However, there are reports (Table 1) suggesting their potential biomarker roles in prostate cancer. In Table 1, the articles comparing the expression of laminins in healthy and cancer tissues are mainly included. The most significant change is the loss of the hemidesmosomal laminin-332 (see further text) in invasive prostate carcinoma, that is, its LAMA3, LAMB3, and LAMC2 subunits, which might be epigenetically silenced in prostate cancer [185].

Along with ECM glycoproteins, proteoglycans were also studied for their biomarker potential in prostate cancer (Table 1). An article that analyzed proteoglycan expression in prostate cancer tissues found extensive changes in proteoglycan expression in prostate tumors, with a decrease in decorin and lumican expression and an increase in syndecan-1 and glypican-1 expression in tumor stroma and their disappearance in tumor epithelial cells [186].

Finally, the biomarker potential of collagen in prostate cancer is also presented in Table 1. In a recent study, by using second-harmonic generation imaging and genotyping of ECM around prostate cancer, the authors showed that the changes in content, orientation, and type of collagen varied with the prostate cancer Gleason grade. Moreover, the ability of the collagen content orientation to predict clinically significant prostate cancer has been demonstrated since there were clear differences between collagen orientation and type in normal and cancer tissues [187]. This would suggest the involvement of changed collagen features in prostate cancer.

**Table 1:** Examples of the ECM genes and proteins with biomarker potential in prostate cancer. Core matrisome genes/proteins were considered. Additionally, MMPs, ADAMs, LOXs, and CTSs from the ECM regulators category are included. Collagen synthesis and breakdown intermediates are omitted from this table and are discussed in the text. CT, cancer tissue; M, metastases; B, blood; S, serum; P, plasma; U, urine; EVs, extracellular vesicles; D, diagnostic; PRO, prognostic; PRE, predictive biomarker; R, biomarker of prostate cancer risk; RS, risk stratification biomarker.

Gene/Protein	ECM Category	Type of Molecule Studied	Compartment Used	Biomarker Type	References
Type I collagen	Collagen	mRNA and protein	CT	D	[188]
Type III collagen	Collagen	mRNA, protein, and autoantibodies	CT, S	D	[188,189]
Type VII collagen	Collagen	Protein	CT	D	[190]
Type VIII collagen	Collagen	Protein	S	D	[191]
Type XX collagen	Collagen	Protein	S	D	[192]
Type XXIII collagen	Collagen	Protein	CT, M, U	D, PRO	[193]
COL4A1 (collagen type IV alpha 1 chain)	Collagen	mRNA	CT, M	D, RS	[194]
COL4A6	Collagen	mRNA	CT	D, PRO, RS	[195,196]
COL10A1	Collagen	mRNA and protein	CT	D, PRO	[197]
ASPN (asporin)	Proteoglycans	Gene, mRNA, and protein	CT, B	D, PRO	[198,199,200,201,202]
BGN (biglycan)	Proteoglycans	mRNA and protein	CT	D, PRO, RS	[199,203,204]
ESM1 (endocan)	Proteoglycans	Protein	CT, S	D, PRO, RS	[205,206,207]
FMOD (fibromodulin)	Proteoglycans	Gene, mRNA, and protein	CT	D, PRO, RS, R	[208,209,210,211]
HSPG2 (perlecan)	Proteoglycans	Protein	CT, S, U	D	[212,213]
SPOCK1 (testican-1)	Proteoglycans	mRNA and protein	CT	D, PRO, RS	[214,215,216]
SPOCK3 (testican-3)	Proteoglycans	mRNA	CT	D, PRO, RS	[217,218]
VCAN (versican)	Proteoglycans	mRNA and protein	CT	D, PRO, RS	[219,220]
ADIPOQ (adiponectin)	Glycoproteins	Gene and protein	CT, S	D, PRO, RS, R	[221,222,223,224,225,226,227,228,229,230]
COMP (cartilage oligomeric matrix protein)	Glycoproteins	mRNA and protein	CT, M, B	D, RS	[202,231,232]
CTHRC1 (collagen triple helix repeat containing 1)	Glycoproteins	mRNA and protein	CT	PRO	[233]
CYR61 (cysteine-rich heparin-binding protein 61)	Glycoproteins	mRNA and protein	CT, S	D, PRO, PRE, RS	[162,163,164,165,234]
DSPP (dentin sialophosphoprotein)	Glycoproteins	mRNA and protein	CT, S	D, RS	[235,236]
EDIL3 (EGF like repeats and discoidin domains 3)	Glycoproteins	Protein	CT, S	D, RS	[237]
EFEMP1 (EGF containing fibulin ECM protein 1)	Glycoproteins	mRNA, protein, and DNA methylation	CT, S, U	D, RS	[238,239,240]
EFEMP2	Glycoproteins	mRNA	CT	D	[216]
FBLN1 (fibulin 1)	Glycoproteins	mRNA and protein	CT	D	[216]
FBLN5	Glycoproteins	mRNA and protein	CT	D	[216]
FBN1 (fibrillin 1)	Glycoproteins	Protein	U	D, RS	[241]
FGG (fibrinogen gamma chain)	Glycoproteins	Protein	S, U	D	[242,243]
FN1 (fibronectin 1)	Glycoproteins	Protein	B	D	[169]
HMCN2 (hemicentin 2)	Glycoproteins	Protein	U	D, RS	[241]
IBSP (integrin-binding sialoprotein)	Glycoproteins	mRNA and protein	CT, M, S	D, PRO, RS	[235,244,245,246,247,248,249,250,251]
IGFBP1 (insulin like growth factor binding protein 1)	Glycoproteins	Gene and protein	S, P	PRO, RS, R	[252,253,254,255,256,257]
IGFBP2	Glycoproteins	mRNA and protein	CT, S	D, PRO, PRE, R?	[161,258,259,260,261,262,263,264,265,266,267]
IGFBP3	Glycoproteins	Gene and protein	CT, S, P, U	D, PRO, RS, R	[268,269,270,271,272,273,274,275,276,277] [261,278,279,280,281,282,283]
IGFBP6	Glycoproteins	Protein	S	D	[284]
LAMA1 (laminin subunit alpha 1)	Glycoproteins	Protein	CT	D	[285]
LAMA3	Glycoproteins	Protein	CT	D	[285]
LAMA5	Glycoproteins	Protein	CT	D	[286]
LAMB1	Glycoproteins	mRNA	CT, M	D, RS	[194]
LAMB3	Glycoproteins	mRNA and protein	CT	D	[287,288]
LAMC2	Glycoproteins	mRNA and protein	CT	D	[190,287,288]
LRG1 (leucine rich alpha-2-glycoprotein 1)	Glycoproteins	Protein	CT, B, P (EVs), U (EVs)	D, PRO, PRE, RS	[146,147,289,290]
NTN1 (netrin 1)	Glycoproteins	mRNA and protein	CT, P	D	[291,292]
POSTN (periostin)	Glycoproteins	mRNA and protein	CT, M, B, P	D, PRO, RS	[166,167,168,169,170,171,174,175,176,232,293,294]
RELN (reelin)	Glycoproteins	Protein	CT	D, RS	[295]
RSPO3 (R-spondin 3)	Glycoproteins	mRNA	CT	D, PRO	[296]
SLIT2 (slit guidance ligand 2)	Glycoproteins	Protein	CT, M	D	[297]
SPARC (osteonectin)	Glycoproteins	mRNA, protein, and DNA methylation	CT, M	D, PRO, RS	[298,299,300,301,302]
SPON1 (spondin 1)	Glycoproteins	mRNA	CT	D, PRO, RS	[217]
SPON2 (spondin 2)	Glycoproteins	Protein and DNA methylation	CT, S	D, RS	[245,303,304,305,306]
SPP1 (osteopontin)	Glycoproteins	mRNA and protein	CT, M, S, P, U	D, PRO, PRE, RS	[246,275,307,308,309,310,311,312,313] [235,250,314,315,316,317,318]
THBS1 (thrombospondin 1)	Glycoproteins	Protein	CT, S, S (EVs)	D, PRO	[319,320,321,322]
THBS2	Glycoproteins	mRNA and protein	CT	D, PRO	[323]
TNC (tenascin C)	Glycoproteins	Gene and protein	CT	D, PRO, RS	[286,324,325,326,327]
VTN (vitronectin)	Glycoproteins	Protein	CT, S, U	D, RS	[241,328]
WISP1 (WNT1 induced secreted protein 1)	Glycoproteins	mRNA and protein	CT	PRO	[329]
ADAM9 (a disintegrin and metalloproteinase domain 9)	ECM regulators	mRNA and protein	CT	D, PRO	[330,331,332,333]
ADAM10	ECM regulators	Protein	CT	D, RS	[334]
ADAM12	ECM regulators	Protein	B, U	D	[335]
ADAM15	ECM regulators	Protein	CT	D, PRO, RS	[336,337]
ADAM19	ECM regulators	mRNA and protein	CT	D, PRO, RS	[338]
ADAM28	ECM regulators	Protein	CT	D	[339]
CTSB (cathepsin B)	ECM regulators	Gene and protein	CT, S	D, RS, R	[340,341,342,343]
CTSB/CSTA (cystatin A) ratio	ECM regulators	mRNA and protein	CT	D, PRO, RS	[344,345,346,347,348]
CTSD	ECM regulators	Protein and protein activity	CT, M, S	D, PRO, PRE, RS	[176,349,350,351,352,353,354,355]
CTSK	ECM regulators	mRNA and protein	CT, M	D, PRE	[356,357]
CTSL	ECM regulators	Protein	CT	RS	[293]
CTSZ	ECM regulators	mRNA and protein	CT, B	D, PRO	[358,359]
LOX (lysyl oxidase)	ECM regulators	Gene, mRNA, and protein	CT, M	D, PRO, PRE, RS	[182,183,360,361,362]
MMP1 (matrix metalloproteinase 1)	ECM regulators	Protein	CT	D, PRO, RS	[363,364]
MMP2	ECM regulators	Gene, mRNA, protein, and protein activity	CT, S, P, U	D, PRO, RS, R	[365,366,367,368,369,370,371,372,373,374,375]
MMP7	ECM regulators	Gene and protein	S	PRO, PRE, R	[376,377,378]
MMP9	ECM regulators	mRNA, protein, and protein activity	CT, B, P	D, PRO, RS	[178,179,366,369,371,375,379,380,381,382,383,384,385]
MMP11	ECM regulators	Gene and protein	CT	D, PRO, RS	[386,387,388]
MMP13	ECM regulators	Protein	CT	D, PRO, RS	[386,389]

Table 2 presents information on the biomarker potential of single integrin and IAC-related genes and proteins in prostate cancer in a non-exhaustive manner. It is evident that integrin subunits, but also heterodimers, were frequently studied for their biomarker potential. For example, a recent article dealt with the commonly studied αVβ3 and αVβ6 integrins in prostate cancer mouse models and patient-derived xenografts and suggested that αVβ3 integrin may promote a shift in lineage plasticity towards a more aggressive, neuroendocrine (NE) prostate cancer phenotype and might be a potential biomarker of NE differentiation in prostate cancer. However, this was not the case with the integrin αVβ6, which was shown to be confined to the adenocarcinoma of the prostate [390]. Among the most prominent changes in integrin expression in prostate cancer tissue is the loss of the hemidesmosome-related integrin α6β4, which was noticed in early publications [190,391]. Hemidesmosomes are the type of IACs that facilitate the stable adhesion of basal epithelial cells to the underlying basement membrane [392]. An early report suggested that prostate carcinoma cells lacked hemidesmosomal proteins, the integrin α6β4, BP180, LAMC2, and collagen VII, but did express BP230, plectin, and the integrin laminin receptors α3β1 and α6β1 [190]. In addition to integrins and their ligands, many secreted factors belong to the category of ECM proteins and, together with their receptors, are biomarkers for specific subtypes of prostate cancer.

Although many proteins are considered to be part of the human adhesome [12], in this article, the sixty consensus adhesome proteins were considered. Furthermore, since the αVβ5 integrin heterodimer is among the most studied integrins, the additional four proteins (talin 2 (TLN2), filamin A and B (FLNA, FLNB), and plectin (PLEC)) that were shown to be part of its adhesome [14] were analyzed for their potential roles in prostate cancer biomarker discovery. Among adhesome proteins, alpha-actinins belong to the cytoskeletal proteins and show multiple roles in different cell types. ACTN4 was shown to promote the progression of prostate cancer [393]. In a study that analyzed the composition of exosomes in different groups of men with metastatic prostate cancer (untreated, well-controlled with primary androgen deprivation therapy (ADT), and castration-resistant prostate cancer (CRPC) patients), the expression of ACTN4 was shown to be increased in the exosomes of CRPC patients in comparison to the ADT group of patients [394]. Furthermore, filamins are proteins involved in the remodeling of the cytoskeleton and, therefore, play important roles in migration and invasiveness. Moreover, AR is found in a complex with FLNA, and the AR/FLNA complex was suggested to be a target in the prostate cancer microenvironment, for example, by using an AR-derived stapled peptide, which disrupts the AR/FLNA complex assembly [59]. FLNA and FLNB are listed in Table 2 for their biomarker potential in prostate cancer. An interesting early paper showed that localization of FLNA also bears biomarker potential in prostate cancer since cytoplasmic localization of FLNA was shown to correlate with metastasis [395], and potentiation of FLNA nuclear localization was even suggested to enhance the effectiveness of androgen deprivation therapy [396]. Moreover, CpG site hypermethylation of FLNC and some other genes was associated with the prediction of the biochemical, local, and systemic recurrence of prostate cancer [397,398]. Integrin-linked kinase (ILK) [399], plectin (PLEC) [400,401], paxillin (PXN) [402], talin 1 (TLN1) [403,404], and vinculin (VCL) [405] are other adhesome proteins that were implicated in prostate cancer and that showed biomarker potential in this cancer type (Table 2).

**Table 2:** Examples of integrin and IAC-related genes and proteins with biomarker potential in prostate cancer. Integrins and the genes/proteins of the consensus adhesome and the adhesome of the often-studied integrin αVβ5 were considered. CT, cancer tissue; M, metastases; BM, bone marrow; B, blood; S, serum; P, plasma; U, urine; EVs, extracellular vesicles; D, diagnostic; PRO, prognostic; PRE, predictive biomarker; R, biomarker of prostate cancer risk; RS, risk stratification biomarker.

Gene/Protein	Category	Type of Molecule Studied	Compartment Used	Biomarker Type	References
ITGA2 (integrin subunit alpha 2)	Integrin	mRNA and protein	CT, BM, M	D, PRO	[406,407,408]
ITGA3	Integrin	Protein	CT, U (EVs)	D, PRO, RS	[150,409,410]
ITGA5	Integrin	Protein	CT, M	D, RS	[91,411]
ITGA6	Integrin	Protein	CT, BM	PRO, RS	[407,408,409,412,413]
ITGA7	Integrin	Gene and protein	CT	D, PRO, RS	[411,414]
ITGAV	Integrin	Gene and protein	CT, M, U	D, PRO, R, RS	[91,415,416,417]
ITGB1	Integrin	Protein	CT, U (EVs)	D, PRO, RS	[150,412,418]
ITGB4	Integrin	Gene, mRNA, and protein	CT	D, PRO	[391,419,420]
ITGB5	Integrin	Protein	CT, S (EVs)	D, RS	[148,421]
α3β1 integrin	Integrin	Protein	CT	PRO	[410]
α6β4 integrin	Integrin	Protein	CT	D	[190,391]
αVβ5 integrin	Integrin	Protein	CT	PRO, RS	[415]
ACTN1 (actinin alpha 1)	Consensus (cons.) and αVβ5 adhesome	Protein	CT	PRO, RS	[422]
ANXA1 (annexin A1)	Cons. adhesome	mRNA and protein	CT, U	D	[243,420]
FEN1 (flap endonuclease 1)	Cons. adhesome	Protein	CT	D, RS	[423]
FHL2 (four and a half LIM domains 2)	Cons. adhesome	Protein (nuclear)	CT	D, PRO, RS	[424,425]
FLNA (filamin-A)	αVβ5 adhesome	Protein	CT, S	D	[395,426]
FLNA, FLNB correlation	αVβ5 adhesome	Protein	S	D	[427]
ILK (integrin linked kinase)	Cons. adhesome	Protein	CT	D, PRO, RS	[428]
IQGAP1 (IQ motif containing GTPase activating protein 1)	Cons. adhesome	mRNA and protein	CT	D, PRO, RS	[429]
P4HB (prolyl 4-hydroxylase subunit beta)	Cons. adhesome	mRNA	CT	D, PRO, PRE, RS	[430]
PDLIM5 (PDZ and LIM domain 5)	Cons. adhesome	Gene, mRNA, and protein	CT	D, PRO, PRE, RS	[431,432,433]
PLEC (plectin)	αVβ5 adhesome	Protein	CT, M	D	[400]
PXN (paxillin)	Cons. adhesome	Protein	CT, M	D, PRE, RS	[434,435]
SORBS1(sorbin and SH3 domain containing 1)	Cons. adhesome	mRNA	CT	D, PRO	[436,437]
TGM2 (transglutaminase 2)	Cons. adhesome	mRNA and protein	CT	D, PRO, RS	[438,439]
TLN1 (talin 1)	Consensus and αVβ5 adhesome	Protein	CT, M	D, PRE, RS	[404,440]
VCL (vinculin)	Consensus and αVβ5 adhesome	Gene and protein	CT, U	D	[441,442]
ZYX (zyxin)	Cons. adhesome	Protein	CT	D	[170,443]

Finally, the ECM and IACs-related genes/proteins currently being tested in active clinical trials include ANXA3 (ClinicalTrials.gov ID: NCT04825002) within the urinary multimarker sensor. The sensor is based on the measurements of trace amounts of four protein biomarkers from naturally voided urine in men referred with clinical suspicion of prostate cancer who have had no prior prostate biopsy. The goal of the study is to test whether a urinary multimarker sensor would help to avoid unnecessary prostate biopsy while detecting clinically significant prostate cancer. Annexins are ECM-affiliated proteins, and some of them are categorized as the meta-adhesome proteins, and ANXA1 is a part of the consensus adhesome. Furthermore, SLPI (secretory leukocyte peptidase inhibitor), which belongs to the category of ECM regulators, is currently being tested to verify whether an increased SLPI level in the sera may serve as a biomarker of cancer progression (NCT04854343). Further to this, ADIPOQ, leptin, IGF1, and IGFBP3 ECM proteins are tested among the potential biomarkers whose levels are changed upon physical activity and eating patterns that improve the body composition of prostate cancer survivors (NCT03971591).

### ECM- and IACs-Related Genes and Proteins Taking a Part in Prostate Cancer Gene and Protein Signatures with Biomarker Potential

The number of studies that define the gene and protein signatures for diagnosis, prognosis, prediction, and/or risk stratification in prostate cancer has increased rapidly in recent years. The ECM- and IACs-related genes and proteins are frequently found as part of those signatures in prostate cancer (e.g., [217,444,445]). Some examples of prostate cancer radical prostatectomy tissues include the gene expression of *NID1* (nidogen 1) and *COL4A6* (collagen type IV alpha 6 chain) within the immune-related five gene signature for prostate cancer risk stratification and prognosis [446] or genes contributing to the ECM’s connective tissue signature (*COL1A1*, *COL1A2*, *COL3A1*, *LUM* (lumican), *VCAN* (versican), *FN1* (fibronectin 1), *AEBP1* (AE Binding Protein 1), *ASPN* (asporin), *TIMP1* (TIMP metallopeptidase inhibitor 1), *TIMP3*, *BGN* (biglycan), *FMOD* (fibromodulin), and *FLNA*) and their expression related to prostate cancer growth and progression [447]. Other examples include the P-score, which consists of the expression of three genes, among which is *IGFBP3*, and adds to the prognosis for prostate cancer patients [448,449,450]. There are also examples of such studies in the blood [202,451,452,453], EVs [454], and urine [243,455,456] of prostate cancer patients. For example, a study that focused on the clinical validation of a serum protein panel consisting of FLNA, FLNB, and KRT19 (keratin 19) found that their expression and PSA in combination were better than PSA alone in identifying prostate cancer. Moreover, they improved the prediction of high- and low-risk disease and the prediction of cancer versus benign prostatic hyperplasia [457]. Another study created a multivariable model comprising ECM protein concentrations of FN1 (fibronectin 1), LUM, MMP9, THBS1, LGALS3BP (galectin-3-binding protein), and PSA from serum, together with clinical grade group and clinical stage. The proposed model was shown to be a predictor of biochemical recurrence after radical prostatectomy, and it was significantly associated with adverse pathology [458].

However, the most prominent gene and protein signatures for prostate cancer include those contained in commercially available tests like the tissue-based Oncotype DX, Prolaris, Decipher, PORTOS, ProMark, and the blood-based Proclarix. Among other genes, some of these tests contain ECM- and IAC-related genes. For example, Oncotype DX tests the expression of 5 reference genes and 12 cancer-related genes, including *BGN* (biglycan), *COL1A1*, *FLNC*, and *SFRP4* (secreted frizzled related protein 4) [459]. While the reviewed genes and proteins from the first part of Section 6 and Table 1 and Table 2 are investigated mainly in early, initial studies that suggested their biomarker potential, the mentioned tests are more studied. However, these tests were mainly not analyzed in prospective clinical trials and, until now, did not receive Food and Drug Administration approval. Still, they are supported for a subset of patients in the most recent European Association of Urology guidelines [120]. Among the tests, Proclarix (Proteomedix, Schlieren, Switzerland) would be probably the most interesting from the point of view of the ECM- and IAC-related molecules since it is based on the expression of two ECM proteins, THBS1 (thrombospondin-1) and CTSD (cathepsin D), in blood. Together with the total PSA, percentage-free PSA, and patient age, it estimates the likelihood of clinically significant prostate cancer. It is convenient to present the evolution of the potential biomarker signature from the initial studies to its inclusion in one of the relevant prostate cancer guidelines by using the example of Proclarix. Namely, initial papers on the potential of THBS1 and CTSD signature in prostate cancer were published in 2017 [460], 2019 [461], and 2020 [462] and were based on the earlier analyses of the serum proteome in metastatic castration-resistant prostate cancer patients and the PTEN conditional knockout mouse model [463,464]. Those initial articles, based on a retrospective cohort of biobanked serum samples, demonstrated and validated the diagnostic biomarker potential of the mentioned protein signature in prostate cancer. Subsequently, the Proclarix test was shown to be suitable for use in clinical practice [465]. Moreover, Proclarix was evaluated in several further prospective studies [466,467,468,469], which confirmed its utility for reducing the number of biopsies that needed to be performed. In one study, Proclarix detected 100% of clinically significant prostate cancer cases and reduced the need for prostate biopsies by 21.3% and insignificant prostate cancer overdetection by 5.3% [469]. The Proclarix test was even analyzed in a systematic review, which confirmed its potential benefit for a subset of prostate cancer patients [470]. A further study compared Proclarix, PSA density, and the MRI-ERSPC risk calculator to select patients for prostate biopsy after multiparametric magnetic resonance imaging (mpMRI). The study concluded that the MRI-ERSPC risk calculator outperformed PSA density and Proclarix in the overall population; however, Proclarix outperformed in a subset of patients within the Prostate Imaging-Reporting and Data System (PI-RADS) ≤ 3 category [471,472]. Several further studies analyzed the combination of Proclarix with the prostate health index [473,474] and MRI [475] and confirmed its utility. Finally, a very recent article suggested that there was a correlation between Proclarix and the aggressiveness of prostate cancer [476]. Taken together, the current literature on Proclarix seems promising; however, many of the cited studies pointed out that further validation is needed.

Just to briefly mention, DNA methylation signatures were also explored for their biomarker roles in prostate cancer. Namely, the gene encoding HAPLN3 (hyaluronan and proteoglycan link protein 3) ECM proteoglycan was found to be a part of the three-gene DNA methylation biomarker with diagnostic [477,478,479] and prognostic [480] potential in prostate cancer. The mentioned studies analyzed prostate cancer tissues; however, the HAPLN3 DNA methylation from prostate cancer patients’ circulating tumor DNA was also shown to be a part of the three methylated gene signature with potential role in prostate cancer diagnosis and risk stratification [481].

## 7. Conclusions

There is a wide discrepancy between the number of research items on prostate cancer biomarkers and the number of potential molecules and other variables that are used in clinics for prostate cancer biomarker purposes. This is in accordance with some estimates, which say that less than 1% of published cancer biomarkers enter clinical practice [482]. Similar to new drugs, new biomarkers are also subject to rigorous validation before they are introduced into routine clinical care. A very early (2001) article on the phases of biomarker development for early detection of cancer recognized five such phases (preclinical exploratory, clinical assay and validation, retrospective longitudinal, prospective screening, and cancer control) [483], while newer studies (2012) suggested three steps in the development of a candidate biomarker (evaluating analytic validity, clinical validity, and clinical utility) [484,485]. However, many of the potential diagnostic (and other) biomarkers from Table 1 and Table 2 of this article are tested only in the initial, phase 1 (or equivalent) preclinical exploratory studies, which use immunohistochemistry, western blots, mass spectrometry, RT-qPCR, gene-expression profiles, or the levels of circulating antibodies in order to compare the expression of a mRNA or a protein in cancer and non-affected samples or to find correlations with different aspects of a disease. While these data might seem promising at this early stage, many other potential diagnostic (and other) prostate cancer biomarkers have failed to progress through rigorous evaluation [486]. It could be noted that even phase 1 studies are frequently a matter of contradictory reports. There are many reasons behind this, including the use of diverse approaches (studying mRNA in contrast to protein or employing different techniques), different compartments (cancer tissue, plasma/serum, urine), or the molecular, morphological, and clinical heterogeneity of prostate cancer (e.g., [487]), just to name a few. Even when the initial reports on biomarker potential are unison, a significant effort must be made to finish other stages of biomarker development. The reasons for their frequent failure have been a matter of debate for quite some time and are explored in more detail elsewhere [20,482,488]. Briefly, weak clinical performance (low specificity, low sensitivity, low prognostic/predictive value, and information unnecessary for clinical decision-making) and original claims that fail validation because of bioinformatic or pre-, post-, and analytical shortcomings are among the main reasons [488]. The intention of this review article was to summarize the knowledge on the biomarker potential stemming from ECM- and IAC-related molecules and processes and to invite closer inspection. A good example is the Proclarix test, which showed very promising results. Many of the papers reviewed in this article are reminiscent of the initial studies on Proclarix, and their topics might be worth studying further. Hence, in summary, the ECM and IACs seem to contribute significantly to the prostate cancer biomarker discovery, and it might be good to thoroughly re-evaluate their potential.
